# Pitavastatin and Lovastatin Exhibit Calcium Channel Blocking Activity Which Potentiate Vasorelaxant Effects of Amlodipine: A New Futuristic Dimension in Statin’s Pleiotropy

**DOI:** 10.3390/medicina59101805

**Published:** 2023-10-10

**Authors:** Wajid Ali, Niaz Ali, Abid Ullah, Shafiq Ur Rahman, Shujaat Ahmad

**Affiliations:** 1 Department of Pharmacology, Institute of Pharmaceutical Sciences, Khyber Medical University, Hayatabad, Peshawar 25100, Khyber Pakhtunkhwa, Pakistan; dr.wajidali161@gmail.com; 2 Department of Pharmacology, College of Medicine, Shaqra University, Shaqra 11961, Saudi Arabia; 3 Department of Pharmacy, Shaheed Benazir Bhutto University, Sheringal, Dir 18200, Khyber Pakhtunkhwa, Pakistan; abid@sbbu.edu.pk (A.U.); shafiq@sbbu.edu.pk (S.U.R.); shujaat@sbbu.edu.pk (S.A.)

**Keywords:** statins, pitavastatin, lovastatin, amlodipine, verapamil, calcium concentration response curves, left shift, EC_50_, right shift, vasorelaxant

## Abstract

*Background and Objectives*: We have recently reported that Fluvastatin, Atorvastatin, Simvastatin and Rosuvastatin have calcium channel antagonistic activities using rabbits’ intestinal preparations. The current study is focused on the effects of Pitavastatin and Lovastatin for possible inhibition of vascular L-Type calcium channels, which may have vasorelaxant effect(s). Combined effects of Pitavastatin and Lovastatin in the presence of Amlodipine were also tested for vasorelaxation. *Materials and Methods*: Possible relaxing effects of Pitavastatin and Lovastatin on 80 mM Potassium chloride (KCL)-induced contractions and on 1 µM norepinephrine (N.E)-induced contractions were studied in isolated rabbit’s aortic strips preparations. Relaxing effects on 80 mM KCL-induced vascular contractions were further verified by constructing Calcium Concentration Response Curves (CCRCs), in the absence and presence of three different concentrations of Pitavastatin and Lovastatin using CCRCs as negative control. Verapamil was used as a standard drug that has L-Type calcium channel binding activity. In other series of experiments, we studied drug interaction(s) among Pitavastatin, Lovastatin, and amlodipine. *Results*: The results of this study imply that Lovastatin is more potent than Pitavastatin for having comparatively lower EC_50_ (7.44 × 10^−5^ ± 0.16 M) in intact and (4.55 × 10^−5^ ± 0.10 M) in denuded aortae for KCL-induced contractions. Lovastatin amplitudes in intact and denuded aortae for KCL-induced contractions were, respectively, 24% and 35.5%; whereas amplitudes for Pitavastatin in intact and denuded aortae for KCL-induced contractions were 34% and 40%, respectively. A left shift in the EC_50_ values for the statins was seen when we added amlodipine in EC_50_ (Log Ca^++^ M). Right shift for CCRCs state that Pitavastatin and Lovastatin have calcium channel antagonistic effects. Lovastatin in test concentration (6.74 × 10^−7^ M) produced a right shift in relatively lower EC_50_ (−2.5 ± 0.10) Log Ca^++^ M as compared to Pitavastatin, which further confirms that lovastatin is relatively more potent. The right shift in EC_50_ resembles the right shift of Verapamil. Additive effect of Pitavastatin and Lovastatin was noted in presence of amlodipine (*p* < 0.05). *Conclusions*: KCL (80 mM)-induced vascular contractions were relaxed by Pitavastatin and Lovastatin via inhibitory effects on L-Type voltage-gated calcium channels. Lovastatin and Pitavastatin also relaxed Norepinephrine (1 µM)-induced contractions giving an insight for involvement of dual mode of action of Pitavastatin and Lovastatin.

## 1. Introduction

Statins are globally used for the treatment of hypercholesterolemia. Statins inhibit cholesterol synthesis by binding competitively and reversibly with the HMG-CoA reductase enzyme [1]. Statins also upregulate LDL receptors which increase serum LDL clearance [2]. In 1978, Alberts, Chen and associates isolated Lovastatin from *Aspergillus terrus* at Merck research laboratory. It was used clinically in 1987 [3,4] In 1997, Mevastatin was isolated from *Penicillium citrinum* for the first time in Japan. Simvastatin a synthetic derivative of Lovastatin was developed in 1988. Several statins such as Fluvastatin in 1991, Atorvastatin in 1997, Cervistatin in 1998, Rosuvastatin in 2003, and Pitavastatin in 2011 were followed by their ancestor statins. Adverse effects were reported, particularly severe rhabdomyolysis that caused withdrawal of Cervistatin from the market [5].

Statins’ Pleiotropic effects warrant further investigations. In Greek, Pleiotropy is a combination of two words: Pleion means more and Trophos means in multiple ways. Thus statins’ pleotropic effects can be beneficial or harmful [6,7]. These pleiotropic effects can be observed in clinical and preclinical studies. Pravastatin and Rosuvastatin have been shown to decrease creatinine levels in patients with normal and abnormal renal function [8,9]. In a meta-analysis study, it is evident that Rosuvastatin decreases the incidence and mortality of pneumonia [10]. Statins upregulate endothelial nitric oxide synthetase (eNOS) that cause increased production of nitric oxide (NO) [11]. Atorvastatin causes a decrease in CRP levels in patients with acute coronary syndrome [12]. Investigation regarding cell lines of vascular tissue reveals that statins increase the expression of calcium channels [13]. Recently we have reported the calcium channel’s antagonistic effects of some of the statins in intestinal preparations [14]. We have also translated that the vasorelaxant effects of Simvastatin, Atorvastatin, Rosuvastatin, and Fluvastatin follow inhibition of voltage gated calcium channels in the isolated aortic strips preparations [15,16]. Thus, the current work is an extension of our project to study the possible vasorelaxant effects of Pitavastatin and Lovastatin. This is because Pitavastatin and Lovastatin were not tested early by our research group. Thus, the aim of the study to determine possible inhibitory effects of Pitavastatin and Lovastatin on voltage-gated calcium channels using isolated aortic strips preparations.

Besides treating hypercholesterolemia with statins, patients are also treated for their concomitant ailments that include antihypertensives, antianginals, and drugs for myocardial infarction. Therefore, the most concomitantly used drugs for management of these diseases are calcium channel blockers, beta-blockers, and angiotensin receptor blockers [17]. Thus, our study is also aimed to investigate possible drug–drug interactions between Pitavastatin, Lovastatin, and amlodipine, a standard calcium channel blocker.

## 2. Materials and Methods

### 2.1. Study Setting

The study was carried out at the Department of Pharmacology, Institute of Pharmaceutical Sciences, Khyber Medical University, Peshawar, Pakistan.

### 2.2. Materials

We used analytical-grade chemicals in the experimental work. Acetylcholine and Norepinephrine were procured from BDH, Poole, UK. Pitavastatin was obtained from GENIX Pharma Peshawar, Pakistan. Lovastatin was purchased from ZAFA, Pakistan. Amlodipine was purchased from Ferozson Labs Pvt Ltd., Nowshera, Pakistan. Solutions and suspensions were prepared on the same day of experiments.

### 2.3. Animals

Local-breed rabbits (male and females) were used in the experiments. Their weight was in the range of 2.0–3.5 kg. The animals were housed at the animal house of Khyber Medical University, Peshawar. The animals had free access to water. Rabbits were fasted overnight before the start of experiments. The study protocols were approved by the relevant research board of KMU. Ethical approval was accorded by Ethical Research Board of Khyber Medical University via no: (/IBMS/IRBE/meeting/2022/9303-6).

### 2.4. Data Recording

To record the responses of aortic strips, transducers (Model No: 0225 Pan Lab S1) connected to bridge amplifiers already coupled with a four-channeled power lab (AD Instruments, Sydney, Australia) were used. Isolated vascular responses were recorded in Lab Chart 7 software supplied with the Power Lab (Model No: 4/25 T).

### 2.5. Solutions

Three types of Kreb’s solutions were used. (1) Normal Kreb’s solution consists of ingredients in (mM): NaCl 118.2, KCL 4.7, KH_2_PO_4_ 1.3, MgSO_4_ 1.2, NaHCO_3_ 25.0, Glucose 11.7, and CaCl_2_ 2.5. (2) K-Normal (Ca^++^ free) Krebs’s solution with ingredients in (mM): NaCl 118.2, KCL 4.7, KH_2_PO_4_ 1.3, MgSO_4_ 1.2, NaHCO_3_ 25.0, Glucose 11.7, and ethylenediamine tetraacetic acid (EDTA) 0.1; and (3) K-Rich (Ca^++^ free) Kreb’s solution with ingredients in (mM): (NaCl 50.58, KCL 50, KH_2_PO_4_ 1.26, MgSO_4_ 3.10, NaHCO_3_ 23.8, Glucose 11.1, and EDTA) 0.1. All solutions were freshly prepared before the start of experimental work.

### 2.6. Pitavastatin, Lovastatin, and Amlodipine Effects on KCL (80 mM) Elicited Contractions

Rabbits of either gender were slaughtered, and their abdomens were opened. Their aortae were excised and placed in a petri dish containing Kreb’s normal solution constantly aerated with carbogen gas. Aortic strips were obtained and transferred to another petri dishes containing Kreb’s normal solution constantly aerated with carbogen gas. The aortae were separated from its connective tissues under a dissecting microscope. The aortae were cut into strips (2–3 mm) with a sharp razor blade. Endothelium intact aortic strips were isolated. A moist cotton swab was used for gently stroking through the luminal surface of aortic strips to denude the aortae. Denuding was subsequently verified through the absence of acetylcholine-induced relaxation which happens in endothelium intact aortae. Aortae were placed in tissue organ baths filled with Normal Kreb’s solution, constantly aerated with carbogen (95% O_2_, 5% CO_2_) at 37 ± 1 °C. A baseline tension (2 g) was applied as preload. An incubation period of 1 h was provided. Different molar concentrations of Pitavastatin and Lovastatin were prepared in deionized water. Initially, a solution of KCL (80 mM) was introduced into the tissue organ baths containing both intact and denuded aortae to elicit sustained contractions. Pitavastatin, lovastatin, and Amlodipine were applied in cumulative manner (final bath concentration in range 10^−8^ to 10^−2^ M). A 1 min gap was given between applications of different test concentrations of test samples. Possible effects on KCL-induced contractions were noted as per our reported protocols [15,16,18]. Mean effective concentration (EC_50_) for Pitavastatin, lovastatin, and Amlodipine were noted [14,19,20].

### 2.7. Pitavastatin, Lovastatin, and Amlodipine Effects on N.E (1 µM) Elicited Contractions

Sustained contractions were produced in endothelial intact and denuded aortae using 1 µM norepinephrine. The tissues were stabilized for 40–60 min. Pitavastatin, lovastatin and amlodipine were applied in cumulative manner (10^−8^ to 10^−2^ M) as described above. Effects on the tissues were noted as per reported protocols [15,16,20,21]. Mean effective concentrations (EC_50_) were calculated for Pitavastatin, Lovastatin, and amlodipine. Experiments were repeated 4 times.

### 2.8. Combined Effects of Pitavastatin, Lovastatin, and Amlodipine

In another series of experiments, contractions were produced via the application of KCL (80 mM) and N.E (1 µM) in intact and denuded aortae. First, Amlodipine was applied in its respective EC_50_ concentration. An incubation period of 40 min was given. Then we applied Pitavastatin and Lovastatin in concentrations ranges of (10^−8^ to 10^−2^ M) in cumulative manner. Responses were recorded and mean effective concentrations (EC_50_) were calculated for the combined effects of Pitavastatin, Lovastatin, and Amlodipine in intact and denuded aortae. The experiments were run four times [15,16]. Possible shifts in EC_50_ values were recorded to show drug interactions, if any.

### 2.9. Pitavastatin and Lovastatin Effects on Calcium Concentration Response Curves (CCRCs)

As KCL (80 mM)-induced contractions were relaxed, which implies for potential activity as calcium channel blocker. Thus, we carried out another series of experiments in denuded aortae. More, as Pitavastatin and Lovastatin relaxed the KCL (80 mM) and N.E (1 µM)-induced contractions, so CCRCs were drawn for confirmation of inhibition of vascular voltage gated calcium channels. Briefly, denuded aortic strips were decalcified by subjecting them to a series of washes with K-normal (Ca^++^ free) Kreb’s solution, following washes with K-rich Kreb’s solution. The tissues were constantly aerated with carbogen gas. After a stabilization period of 30–40 min, Control Calcium Concentration Response Curves (CCRCs) were drawn in the absence of Pitavastatin and Lovastatin. Control CCRCs were drawn in calcium range of 1 × 10^−4^–250 × 10^−4^ (Log Ca^++^ Molar Solution). Then, we applied different test concentrations of Pitavastatin and Lovastatin. Again, an incubation period of 1 h was given. CCRCs were again constructed in the presence of different concentrations of Pitavastatin and Lovastatin. In similar fashion, control curves for CCRCs, in absence of verapamil, were constructed. Again, in test concentrations of verapamil CCRCs were constructed after an incubation period of 1 h. Their EC_50_ were calculated and possible shift in EC_50_ were recorded. The experiments were repeated 4 times as per our reported procedures [14,15,20,21,22].

### 2.10. Statistical Analysis

Responses of isolated aortic strip preparations were presented as % of control maximum of (80 mM) KCL-induced contractions. Similarly, responses for NE (1 µM)-induced contractions was plotted on *Y*-axis versus test concentrations of Pitavastatin, Lovastatin, and Amlodipine on *X*-axis. Graph Pad Prism version 8 were used to calculate their respective EC_50_ values. For CCRCs, responses were plotted on *Y*-axis versus its respective log Calcium Molar (M) concentrations on *X*-axis. EC_50_ values were compared versus its respective control EC_50_ using one way ANOVA in Graph Pad Prism version 8 at 95% confidence interval (CI) with *p* < 0.05.

## 3. Results

**Figure 1:**
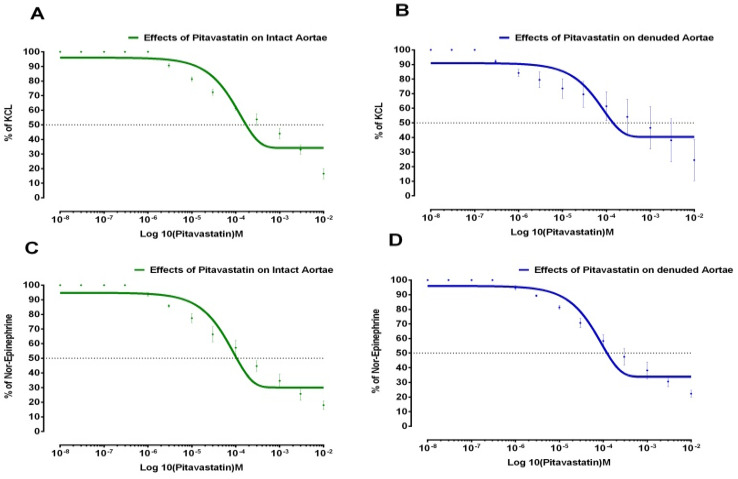
Effects of Pitavastatin on aortic strips in intact and denuded tissues. (**A**) Pitavastatin’s effects on KCL elicited contractions in intact aortic strips. (**B**) Pitavastatin’s effects on KCL elicited contractions in denuded aortic strips. (**C**) Pitavastatin’s effects on Norepinephrine elicited contractions in intact aortic strips. (**D**) Pitavastatin’s effects on Norepinephrine elicited contractions in denuded aortic strips (All values represent the Mean ± SD, *n* = 4, *p* < 0.05).

Effects of Pitavastatin on 1 µM Norepinephrine and 80 mM KCL-induced contractions in isolated aortic strips (both in Intact and Denuded) preparations are shown in Figure 1.

**Figure 2:**
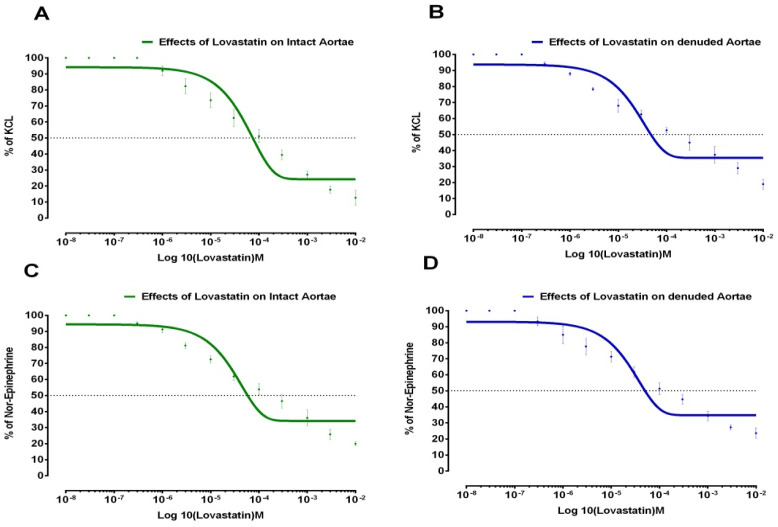
Effects of Lovastatin on aortic strips in intact and denuded tissues. (**A**) Lovastatin’s effects on KCL elicited contractions in intact aortic strips. (**B**) Lovastatin’s effects on KCL elicited contractions in denuded aortic strips. (**C**) Lovastatin’s effects on Norepinephrine elicited contractions in intact aortic strips. (**D**) Lovastatin’s effects on Norepinephrine elicited contractions in denuded aortic strips (All values represent the Mean ± SD, *n* = 4, *p* < 0.05).

**Figure 3:**
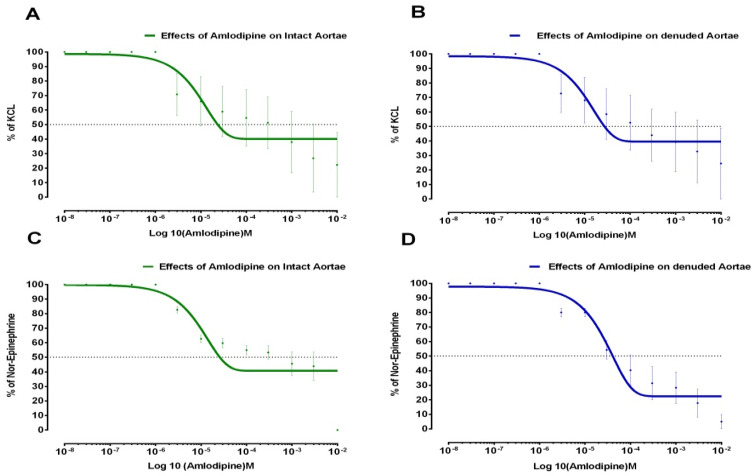
Effects of Amlodipine on aortic strips in intact and denuded tissues. (**A**) Amlodipine’s effects on KCL elicited contractions in intact aortic strips. (**B**) Amlodipine’s effects on KCL elicited contractions in denuded aortic strips. (**C**) Amlodipine’s effects on Norepinephrine elicited contractions in intact aortic strips. (**D**) Amlodipine’s effects on Norepinephrine elicited contractions in denuded aortic strips (All values represent Mean ± SD, *n* = 4, *p* < 0.05).

Similarly, effects of Lovastatin and Amlodipine on (1 µM) N.E and (80 mM) KCL-induced contractions in isolated aortic strips preparations (intact and denuded) are shown in Figure 2 and Figure 3, respectively.

Respective EC_50_ for Pitavastatin, Lovastatin, and Amlodipine in intact and denuded aortae are shown in Table 1. Maximum relaxing effects of Pitavastatin, Lovastatin, and Amlodipine as % of control maximum for 80 mM (KCL) and 1 µM (N.E)-induced contractions are also shown.

It is pointed out that the Lovastatin EC_50_ concentrations for KCL- and NE-induced contractions for intact and denuded aortae are lesser as compared to EC_50_ concentrations for Pitavastatin. This implies that Lovastatin is more potent than Pitavastatin. The vasorelaxant effects of Pitavastatin, Lovastatin, and Amlodipine can be compared. Lovastatin exhibited relatively more potent relaxing effect as compared to Pitavastatin. In case of denuded aortae, the vasorelaxant effects of Lovastatin are almost comparable to the vasorelaxant effect of Amlodipine, a standard vasorelaxant.

The effects of possible drug interactions (in vitro) are presented in Table 2. This shows that EC_50_ for the test statins on KCL- and NE-induced contractions experienced a left shift as compared to statins EC_50_ values when tested alone. The left shift in EC_50_ values implies for additive effect of the statins when tested in presence of Amlodipine, a standard vasorelaxant.

Table 2 represents that the % relaxant effect on N.E (1 µM)-induced contractions and KCL (80 mM)-induced contractions is more as compared to its respective % relaxing effects mentioned in Table 1. This represents the additive effect of Pitavastatin with Lovastatin and Amlodipine as well. These effects are additive, as it does not matter if we use the statins first and then try the amlodipine or amlodipine first, and then study the effects of the test statins.

**Figure 4:**
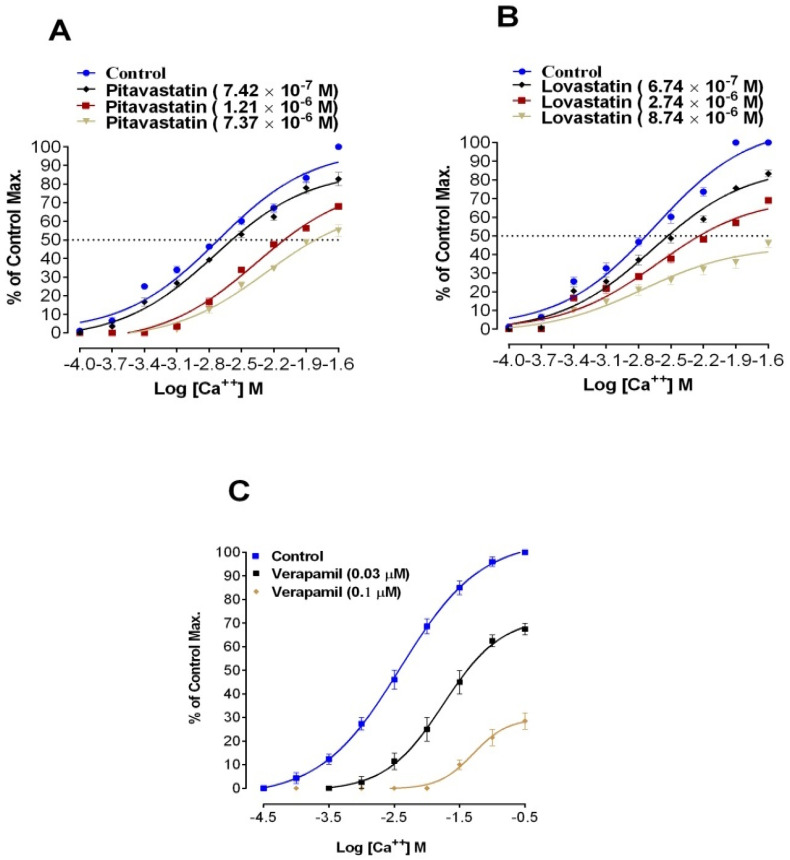
To show the effects of Pitavastatin, Lovastatin, and Verapamil on Calcium Concentration Response Curves. (**A**) CCRCs in the absence and presence of different concentrations of Pitavastatin in aortae. (**B**) CCRCs in the absence and presence of different concentrations of Lovastatin in aortae. (**C**) CCRCs in the presence and absence of various concentrations of Verapamil in aortae (All values are Mean ± SD, *n* = 4).

EC_50_ values for statins, derived from CCRCs (Figure 4) are shown in Table 3. Pitavastatin in concentration (1.21 × 10^−6^ M) caused a right shift in EC_50_ −2.1 ± 0.15 [Log Ca^++^] M vs. its respective control EC_50_ −2.7 ± 0.1 [Log Ca^++^] M. Similarly, Lovastatin in test concentration (2.74 × 10^−6^ M) caused a right shift in EC_50_ −2.3 ± 0.12 [Log Ca^++^] M vs. its respective control EC_50_ −2.7 ± 0.08 [Log Ca^++^] M. This right shift of EC_50_ values by Pitavastatin and Lovastatin resembles the right shift of verapamil, (A standard calcium channel blocker).

## 4. Discussion

As Pitavastatin and Lovastatin relaxed the 80 mM KCL-induced contractions, which implies that these statins may involve inhibition of voltage gated L type calcium channels. More, relaxing effects of Pitavastatin and Lovastatin on 1 µM NE-induced contractions suggest for control on release of calcium from the internal stores through alpha 1 receptor operated calcium channels. Hence, it is argued that statins may have dual mode of action. These benefits can be of interest while managing patients who are hypercholesteremic and hypertensives. The literature suggests that the most common cardiovascular diseases are hyperlipidemia, hypertension, angina pectoris, and myocardial infarction, that sometimes requires practice of polypharmacy besides monotherapy [17,23,24]. Cardiovascular diseases are most commonly observed to occur as comorbid conditions [25]. According to the literature, combination therapy with antihypertensive and antihyperlipidemic drugs provide cardiovascular benefits [26]. The Food and Drug regulatory Authority (FDA) has approved several drugs for treatment of cardiovascular diseases. Statins, beta-blockers, calcium channel blockers, and angiotensin receptor blockers are mostly prescribed drugs [27]. As stated earlier, patients suffering from comorbid conditions such as hypertension and hyperlipidemia are often treated with combination therapy, such as calcium channel blockers and statins [26]. Thus, the importance of additive effects of Pitavastatin and Lovastatin with Amlodipine signify the rationale for possible beneficial effects to control concomitant hypertension. As Pitavastatin and Lovastatin have voltage-gated calcium channel blocking activity, so this could be another reason that best describes the upregulations of calcium channels in the cell lines tissues. However, this upregulation requires time to develop [13]. However, this study is focused on finding the direct instant effect(s) of statins on vascular L-type calcium channels alone and together with amlodipine, a standard calcium channel blocker. In a study conducted by our research group on rabbit’s intestine, it was observed that current statins exhibit antagonistic activity towards voltage-gated calcium channels [14]. In the light of pleiotropic effects of statins and its concomitant use with calcium channel blockers, the possibility of drug–drug interaction between these drugs cannot be set aside. This study showed that Pitavastatin and Lovastatin have inhibitory effects on vascular L-type calcium channels which is proved via a right shift in the EC_50_ for CCRCs. This study also highlights the additive effect of statins with calcium channel blocker amlodipine.

As Pitavastatin and Lovastatin relaxed the KCL- and N.E-induced contractions. Lovastatin is more potent than Pitavastatin. It is evident from the amplitude of % response on KCL (80 mM)-induced contractions which remained up to 24% in intact and up to 35.5% in denuded aortae for lovastatin. While for Pitavastatin its relaxation is up to 34% (intact) and up to 40% (denuded) aortae. These observations are well supported by the values of EC_50_ for Lovastatin and Pitavastatin as shown in Table 1. The relaxing amplitudes of the controlled % response on N.E (1 µM)-induced contraction of Pitavastatin and Lovastatin remains close to each other (Table 1), as N.E mobilizes the Ca^++^ from internal stores and external environment as well. Lovastatin and Pitavastatin’s effects on KCL (80 mM) and N.E (1 µM) are significant. This suggests that statins induce relaxation in vascular strips preparations through multiple ways. When we performed Swiss target prediction, it was evident that Lovastatin has strong interactions with the Norepinephrine transporter, whereas Pitavastatin does not interact with Norepinephrine transporters [28]. This suggests that Lovastatin is more potent and may work through other possible mechanisms in addition to calcium channel blocking activity and on the release of calcium through alpha 1 receptor operated channels.

To rule out any difference in the effects of statins as a target or precipitant drugs, a statins and amlodipine combination was designed accordingly. A left shift in combined EC_50_ values can be observed as compared to EC_50_ values for individual statins. Meanwhile, a left shift in EC_50_ (when used in combination with amlodipine) implies that Pitavastatin, Lovastatin, and Amlodipine have additive effects.

The relaxing effects on KCL-induced contractions require further confirmations for the inhibition of voltage-gated calcium channels. For confirmation of statin’s effects on vascular L-type calcium channels, CCRCs were constructed both in the absence (Control Curve) and presence of different concentrations of tested statins (Pitavastatin and Lovastatin). A right shift in the EC_50_ (Log Ca^++^ M) for CCRCs implies that Pitavastatin and Lovastatin have calcium channel blocking activity. Lovastatin in test concentration 6.74 × 10^−7^ M produced right shift in EC_50_ with a relatively lower EC_50_ (−2.5 ± 0.10) Log Ca^++^ M suggesting that Lovastatin is more potent than Pitavastatin.

EC_50_ right shift in CCRCs endorses the L-type calcium channel inhibitory effects for the tested statins. This finding is also supported by the study findings of one of our other research groups working on isolated rabbits’ jejunal preparations [14]. It is to point out that smooth muscles’ contractions are mediated by voltage-gated calcium channels and receptor-operated calcium channels. Thus, the relaxing effects of Pitavastatin and Lovastatin through inhibition of calcium channels and on N.E-induced contractions can be of leading importance in stress-caused vasoconstriction or in situations where an adrenaline surge is noticed. When we obtained an early Swiss Target Prediction [29,30] for Pitavastatin and Lovastatin, it was observed that Lovastatin had more affinity for N.E transporters besides having affinity for Neurokinin-2 receptors and Dopamine receptors. This means that other modes of involvement cannot be ruled out besides its calcium antagonistic effect on voltage gated calcium channels, that we confirmed in our current study. Stimulation of Norepinephrine transporter causes an increased reuptake of norepinephrine into the presynaptic cleft leading to action termination of Norepinephrine and subsequent relaxation of vascular tissues. These can only be confirmed once we translate the sites of target prediction studies into an in vivo model, that requires further work. However, this study suggests that Pitavastatin and Lovastatin have direct inhibitory effects on the calcium channels, besides their inhibitory effects on the release of calcium through receptor operated calcium channels. More, Lovastatin is more potent than Pitavastatin, as it may involve multiple pathways besides standard calcium channel-blocking activity on L-Type calcium channels.

This study observed the direct actions of test statins on vascular aortic strips. There is some meta-analysis which shows few blood pressure lowering effects [31]. This may be attributed to the autoregulation by body compensatory mechanisms [25]. So, a clinical study in the presence of autoregulation inhibitors should be conducted to find out the vasorelaxant effects of Pitavastatin and Lovastatin. As statins are currently prescribed for their lipid-lowering action alone or in combination with other vasorelaxants, therefore, considering the additive effects of statins, we recommend a prospective randomized controlled clinical trial. This will help in selection of right dose of the statins for its possible antihypertensive effects besides its simultaneous cholesterol-lowering dose.

## 5. Conclusions

The relaxing effects of Pitavastatin and Lovastatin on high molar KCL-induced contractions are predominantly mediated via the L-Type calcium channels’ inhibition. Pitavastatin and Lovastatin also relaxed the Norepinephrine-induced contractions, pointing towards their dual mode of action. More, Lovastatin is more potent than Pitavastatin, which has additive or potentiating effects in the presence of test statins or in the presence of amlodipine. 

### Recommendations

We did not study the effects of Lovastatin and Pitavastatin in perspective of Swiss Target Prediction, which require further work for confirmation of involvement of some other mechanisms beside its calcium channel blocking activity that we explored in this research. Further studies should also be performed to find out the effects of tested statins on calcium internal release to establish its detailed mechanisms.

## Figures and Tables

**Table 1:** To show the relaxing effects of Pitavastatin, Lovastatin, and Amlodipine with their respective EC_50_ values (All values are Mean ± SD, *n* = 4).

Drugs	Aortae Status	% of KCL (Control Max)	% of NE (Control Max)	EC_50_ ± SD KCL-Induced (Molar)	EC_50_ ± SD NE-Induced (Molar)
Pitavastatin	Intact	34	30	1.76 × 10^−4^ ± 0.28	1.05 × 10^−4^ ± 0.20
	Denuded	40	33	1.41 × 10^−4^ ± 0.07	1.16 × 10^−4^ ± 0.17
Lovastatin	Intact	24	34.7	7.44 × 10^−5^ ± 0.16	5.68 × 10^−5^ ± 0.10
	Denuded	35.5	34	4.55 × 10^−5^ ± 0.10	4.90 × 10^−5^ ± 0.08
Amlodipine	Intact	40	40.5	2.3 × 10^−5^ ± 1.11	2.52 × 10^−5^ ± 1.81
	Denuded	39	22.9	3.29 × 10^−5^ ± 1.69	3.83 × 10^−5^ ± 1.52

**Table 2:** To show the combined effects of Pitavastatin, Lovastatin, and Amlodipine with their corresponding EC_50_ values (all values are mean ± SD, *n* = 4).

Drugs	Aortae Status	% of KCL (Control Max)	% of NE (Control Max)	EC_50_ KCL-Induced (Molar)	EC_50_ NE-Induced (Molar)
Amlodipine + Pitavastatin	Intact	14.6	4.73	6.11 × 10^−5^ ± 0.19	3.10 × 10^−6^ ± 1.99
	Denuded	14.9	11.2	1.36 × 10^−5^ ± 0.04	1.52 × 10^−6^ ± 0.99
Amlodipine + Lovastatin	Intact	5.26	6.60	8.11 × 10^−6^ ± 0.02	2.58 × 10^−5^ ± 0.08
	Denuded	9.01	10.3	5.21 × 10^−6^ ± 0.01	1.15 × 10^−5^ ± 0.04
Pitavastatin + Amlodipine	Intact	6.07	14.9	4.84 × 10^−6^ ± 0.01	1.97 × 10^−5^ ± 0.01
	Denuded	6.33	9.28	2.17 × 10^−5^ ± 0.08	3.02 × 10^−5^ ± 0.70
Lovastatin + Amlodipine	Intact	9.28	11.4	1.54 × 10^−5^ ± 0.06	1.06 × 10^−5^ ± 1.15
	Denuded	9.81	12.2	8.95 × 10^−6^ ± 0.03	2.28 × 10^−5^ ± 0.70

**Table 3:** To show the right shift EC_50_ of Pitavastatin, Lovastatin, and Verapamil on Calcium Concentration Response Curves (All values are Mean ± SD, *n* = 4).

Statins	CCRCs Specifications	EC_50_ Log [Ca^++^] M
Pitavastatin	Control	−2.7 ± 0.1
	Test Concentration (7.42 × 10^−7^ M)	−2.6 ± 0.14 **
	Test concentration (1.21 × 10^−6^ M)	−2.1 ± 0.15 **
Lovastatin	Control	−2.7 ± 0.08
	Test Concentration (6.74 × 10^−7^ M)	−2.5 ± 0.10 **
	Test concentration (2.74 × 10^−6^ M)	−2.3 ± 0.12 **
Verapamil	Control	−2.4 ± 0.11
	Test Concentration (0.03 µM)	−1.41 ± 0.22 **
	Test concentration (0.1 µM)	−0.70 ± 0.2 **

(**) shows highly significant values vs. Standard (Verapamil) using one-way ANOVA, *p* < 0.05.

## Data Availability

Power lab data sheets analyzed during this study are available with the corresponding author on formal/reasonable request.

## Abbreviations

Ach	Acetylcholine
CVD	Cardiovascular diseases
CCRCs	Calcium Concentration Response Curves
EC50	Median effective concentration
FDA	Food and Drug Administration
KCL	Potassium chloride
KMU	Khyber Medical University
mM	Millimole
N.E	Norepinephrine
NET	Norepinephrine Transporter
